# The Law Challenged and the Critique of Identity with Emmanuel Levinas

**DOI:** 10.1007/s11196-021-09845-7

**Published:** 2021-05-30

**Authors:** Susan Petrilli

**Affiliations:** 1 Università degli Studi di Bari Aldo Moro, Via M. Garruba, n. 6, 70122 Bari, Italy; 2 The University of Adelaide, Adelaide, SA Australia

**Keywords:** Alterity, *Autrui*, Ethics, Face, Fear, Identity, Justice, New humanism, Language, Other, Proximity, Responsibility, Semiotics, Semioethics, Sign, Subjectivity, Substitution, World, Writing

## Abstract

Identity as traditionally conceived in mainstream Western thought is focused on theory, representation, knowledge, subjectivity and is centrally important in the works of Emmanuel Levinas. His critique of Western culture and corresponding notion of identity at its foundations typically raises the question of the other. Alterity in Levinas indicates existence of something on its own account, in itself independently of the subject’s will or consciousness. The objectivity of alterity tells of the impossible evasion of signs from their destiny, which is the other. The implications involved in reading the signs of the other have contributed to reorienting semiotics in the direction of *semioethics*. In Levinas, the I-other relation is not reducible to abstract cognitive terms, to intellectual synthesis, to the subject-object relation, but rather tells of involvement among singularities whose distinctive feature is alterity, absolute alterity. *Humanism of the other* is a pivotal concept in Levinas overturning the sense of Western reason. It asserts human duties over human rights. Humanism of alterity privileges encounter with the other, responsibility for the other, over tendencies of the centripetal and egocentric orders that instead exclude the other. Responsibility allows for neither rest nor peace. The “properly human” is given in the capacity for absolute otherness, unlimited responsibility, dialogical intercorporeity among differences non-indifferent to each other, it tells of the condition of vulnerability before the other, exposition to the other. The State and its laws limit responsibility for the other. Levinas signals an essential contradiction between the primordial ethical orientation and the legal order. Justice involves comparing incomparables, comparison among singularities outside identity. Consequently, justice places limitations on responsibility, on unlimited responsibility which at the same time it presupposes as its very condition of possibility. The present essay is structured around the following themes: (1) Premiss; (2) Justice, uniqueness, and love; (3) Sign and language; (4) Dialogue and alterity; (5) Semiotic materiality; (6) Globalization and the trap of identity; (7) Human rights and rights of the other: for a new humanism; (8) Ethics; (9) The World; (10) Outside the subject; (11) Responsibility and Substitution; (12) The face; (13) Fear of the other; (14) Alterity and justice; (15) Justice and proximity; (16) Literary writing; (17) Unjust justice; (18) Caring for the other.

## Premiss

Let me begin by premising that this is not an essay *on* Emmanuel Levinas, nor is it classifiable under the formula “What has not yet been said about Levinas”. Even less so are we concerned here specifically with “Levinas’s Judaism,” with his “Judaic roots”. Emmanuel Levinas (1906–1995) rejected the appellative “penseur juif”. Just as he did not consider himself a “religious thinker” if this expression implies that his philosophy is based on a revealed truth, in the same way he did not consider himself a Jewish thinker. He conceived his philosophical meditations in terms of critique and not of obedience to tradition and authority, whether Jewish or otherwise. In response to a question from François Poirié (“Entretiens” [99]), asking him whether it was correct to present him as a “penseur juif” and whether for him “cela a un sens”, Levinas clarified as follows:

Me considérer comme un penseur juif est una chose que ne me choque nullement en soi, je suis Juif. […] Mais je proteste contre cette formule quand on entend par-là quelqu’un qui ose des rapprochements entre concepts bases uniquement sur la tradition et les textes religieux sans se donner la peine di passer par la critique philosophique. (Levinas [99], p. 110)

And to avoid misunderstanding, Levinas further explained that no doubt suggestions may be found in Holy Scripture and that it is possible to take a religious text, such as the Bible, as the starting point for analysis and research. But philosophical truth cannot be based on the authority of the biblical text, or for that matter on the authority of any other religion:

Une vérité philosophique ne peut pas se baser sur l’autorité du verset. […] Mais le verset peut permettre la recherche d’une raison. […] Il m’irrite quand on insinue que j’epreuve par le verset, alors que parfois je cerche par la sagesse ancienne et j’illustre par le verset, oui, mais je ne prouve pas par le verset. (*ibid*., p. 111)

Continuing his exchanges with Poirié, Levinas adds that it makes sense to refer his philosophy to the Jewish religion, but only under precise conditions. The question calls to be framed differently in order to underline the relationship between sacred scripture and thought, reason:

Si vous me posiez la question autrement: est-ce que vous pensez que la *Bible* est essentielle à la pensée? Je répondrais: oui! (*ibid*.)

In any case what Levinas adds immediately after is of particular interest from the point of view of the topic treated in the present essay. He meditates on the I-other relationship, the question of responsibility for the other, the importance of recognizing the other, greater he believes than to recognize the object itself. In Levinas’s view knowledge of the object presupposes recognition of the other. Such a thing as objectivity could not subsist without keeping account of the existence of others: “l’homme est celui qui cerche la vérité”. And he traces a philosophical dimension of Sacred Scipture in the capacity to signify beyond the verse, the letter, beyond plain meaning, beyond the word as an instrument of knowledge, functional to maintaining the objective and political order (see Levinas, *L’au-delà du verset*, [47, 48]). The vocation of language for the other is particularly evident in the language of Sacred Scripture:

la *Bible* nous enseigne que l’homme est celui qui aime son prochain et que le fait d’aimer son prochain est une modalité de la vie sensée ou pensée aussi fondamentale—je dirai plus fondamentale—que la connaissance de l’object et que la vérité en tant que connaissance d’objects. (Levinas [99], p. 113)

Levinas thus continues to explain his relationship to religion:

Dans ce sens là, si on estime que cette deuxième manière d’entendre la pensée est religieuse, je suis penseur religieux! (*ibid*.)

At this point Levinas reiterates what his writings emphasize repeatedly throughout (cf. *À l’heure de nations* [53], ch. 8), that Europe is the Bible and the Greeks together:

Je pense que l’Europe, se sont la *Bible* et les Grecs, mais c’est la *Bible* aussi et qui rend nécessaire les Grecs. (Levinas [99], p. 113).

Here then, in Levinas’s own words, we read what I will attempt to illustrate in the present essay:

Parce-que l’humain commence, ou si vous voulez le sujet commence, à partir de ça relation [the relation to the other], de son obligation à l’égard d’autrui (*ibid*., p. 113).

As utopic, naïve, even optimistic as Levinas’s view of the world might seem, let us remember that his “humanism” was conceived and consolidated in the “inferno” of “Stalag 1492”: “la scoperta dell’umanità nell’inferno dello Stalag 1492”—the discovery of humanity in the inferno of Stalag 1492—is the subtitle of a book by Bernhard Casper titled *Emmanuel Levinas* [25].

## Justice, Uniqueness, and Love

In what follows I mainly consider the contribution that may come to our topic from Emmanuel Levinas, but also proceeding beyond Levinas. I attempt a dialogue with Levinas keeping account of the current historical situation as well as of other texts, other studies in the philosophical tradition relating to our concerns here. In addition to this, I also valorize the contribution that may come to philosophical reflection from the science of signs, semiotics–specifically, for what concerns my interests in this essay, “semiotics of interpretation,” “semiotics of significance,” “global semiotics” ([86, 94, 95, 97], Calabrese et al. [23]).

Under this aspect, I refer the question of the relationship between respect of the law, the rights of identity and the rights of others specifically to the works of Levinas, thus addressing as such is treated as a properly philosophical question. As anticipated, we know that Levinas did not neglect the possibility of contributions from the Jewish religion, with special reference to the Bible for a better understanding of what is at stake. And given that the whole issue doubtlessly involves interpreting texts, languages, communication and inevitably signs, contributions can also be applied and developed from semiotics, the general science of signs.

We refer to representatives of a given tradition in semiotic studies, knowing full well that there are others just as valid and significant as those mentioned: scholars such as Juri Lotman, Paul Ricoeur and Algirdas Greimas. To investigate eventual contributions to our theme from such scholars is another possible research itinerary, no doubt important to develop. Nor do I exclude the possibility of working in this direction myself in relation to my own interests and special “bend,” introduced in semiotics with Augusto Ponzio, denominated *semioethics* ([70, 71, 93, 96]), pp. 184–186. Nonetheless, my immediate task in the present context is not to consider “other semioticians”—whom I would not even connote in such terms (that is, “other semioticians”) with respect to the *philosopher* Emmanuel Levinas, considering that Levinas would not have defined himself as a semiotician, and nor do I, but to focus on Levinas’s philosophical meditations in relation to the theme proposed.

What I find particularly interesting in relation to the Levinasian conception of the law is the claim that the law arises as a function of the other, as a function of original responsibility for the other. In other words, the law does not arise from *fear of* the other (Hobbes), but from *fear for* the other, from the relationship of unindifference, of inevitable implication, involvement, of responsibility for the other’s situation, for the circumstances that concern him/her, for the life of others; ultimately, the law arises from the demand of love and care for the other.

That the law, as it has manifested itself historically, can fail to recognize, even disregard the needs of others, that the law can oppose the life of others, thwart it, to the point even of demanding the extermination of lives, not only imposing death of the individual but full-scale genocide, is something we know full well and cannot deny (cf. [2]). Levinas was certainly among those who knew this better than others. What Franz Kafka prefigures in “Before the law” (in *The Trial*, 1925) is achieved well beyond any literary imagination on the dystopian and arbitrary use of the law. Who was to understand this better than Levinas himself? The injustice of law is the object of reflection throughout the whole course of his writings through to his essay “Les droits de l’homme et les droits d’autrui” [50]. With this title in itself Levinas already underlines the fact that the law is often misused to safeguard self-interest, the interests of identity, affiliation, wherewith trampling the rights of others.

But in spite of what he had suffered personally as a result of his experience with Nazi extermination camps—or perhaps better, precisely because of this experience, and certainly based on this experience, Levinas believed that the law arises from the situation of *responsibility for* the other. The law is imposed by responsibility for the other, which means to say that responsibility for the other calls for the law. The allusion here is to a form of responsibility that is inscribed in the face of the other, unlimited responsibility as Mikhail Bakhtin also calls it (see [22, 80, 81, 85]), responsibility demanded of me before it is inscribed in the letter, *avant la lettre*, before the word. This demand for responsibility towards the other comes from the face of the other, its nudity, from the condition of exposition, from the face’s vulnerability. Injustice signifies that the law is perfectible and that “perfectibility” of the law cannot but consist in recognizing the rights of others. And recognition of the rights of others is the condition for recognition of one’s own rights.

Responsibility leads to justice and justice leads to the theoretical. Justice calls for judgement, comparison, equity and objectivity and is the basis of the theoretical generally, of knowledge, verification, and evaluation. As such justice is the basis of philosophy and philosophy tells of wisdom that arises from the depths of unindifference, charity and love (see “Philosophy, Justice and Love” [22], in Levinas, *Entre nous. Essai sur le penser-à-l’autre* [55], Eng. trans. pp. 103–122). In the words of Levinas:

From the start the encounter with the Other is my responsibility for him. That is the responsibility for my neighbor, which is, no doubt, the harsh name for what we call love of one’s neighbor; love without Eros, charity, love in which the ethical aspect dominates the passionate aspect, love without concupiscence. (*ibid*., p. 103-104)

And here Levinas makes an important clarification concerning the concept of love:

I don’t very much like the word love, which is worn-out and debased. Let us speak instead of the taking upon oneself of the fate of the other. (*ibid*., p. 104)

But with the “first comer” there is always a “third party,” thus the need for justice, which Levinas explains in tones almost paradoxical, given the inevitable implication of justice with the unique, dialogically interrelated with the concepts of equity and objectivity. À propose the “first comer,” continuing with Levinas:

That is the “vision” of the Face, and it applies to the first comer. If he were my only interlocutor, I would have nothing but obligations! But I don’t live in a world in there is but one single “first comer”; there is always a third party in the world: he or she is also my other, my fellow. Hence is it important for me to know which of the two takes precedence. Is the one not the persecutor of the other? Must not human beings, who are incomparable, be compared? Thus justice, here, takes precedence over the taking upon onself of the fate of the other.

I must judge, where before I was to assume responsibilities. Here is the birth of the theoretical; here the concern for justice is born, which is the basis of the theoretical. But it is always starting out from the Face, from the responsibility for the other that justice appears, which calls for judgement and comparison, a comparison of what is in principle incomparable, for every being is unique; every other is unique. In that necessity of being concerned with justice [the] idea of equity appears, on which the idea of objectivity is based. At a certain moment, there is a necessity for a “weighing,” a comparison, a pondering, and in this sense, philosophy would be the appearance of wisdom from the depths of that initial charity; it would be—and I’m not playing on words, the wisdom of that charity, the wisdom of love. (*ibid*.)

The Greek tradition demands that we compare the incomparable; and the Bible tells us that comparison of incomparables, of singularities through the universalization of concepts and laws is justified and mitigated by mercy and compassion for the other, making for a system that can be perfected on the basis of love and care for the other (see [78]), love for the other where the ethical dimension prevails over the passionate (see “The Other, Utopia and Justice”, 1988, *ibid*., pp. 223–234).

## Sign and language

Thanks to its perspective on signs, at once global and detotalizing, semiotics as shaped especially by the research of Thomas A. Sebeok (1920–2001), that is, “global semiotics” [116, 117]—the arrival point, however provisional, of a trend in semiotic studies that has been emerging in modern times, delineated by such figures as Charles S. Peirce (1839–1914), Victoria Lady Welby (1837–1912) and Charles Morris (1901–1975) and, independently from these scholars, also by Mikhail M. Bakhtin (1895–1975)—offers a significant platform for the critique of identity and its signs [91, 92], Petrilli and Ponzio [65], and “A Tribute to Thomas A. Sebeok” [29], pp. 307–330, see also Sebeok et al. [114]. Our issue is not with identity *tout court*, but with what reading Charles Morris may be tagged “closed identity”, with reference to the single human subject “closed self,” a pre-constituted and egocentric ontological entity closed to the other, with claims to self-sufficiency, independency, individual freedom characteristic of such tendencies as those subsumed under the categories of anthropocentrism, ethnocentrism, glottocentrism with their monological and monolinguistic limitations (see [63–66, 77]: 97–150), in the Athanor series, see the volume titled, *Maestri di segni e costruttori di pace* [88]. Thus oriented, semiotics has a philosophical foundation—our core interest in this text—particularly in those trends in contemporary philosophy that are oriented in a critical sense towards Western thought, our first interest here being philosophical research as inaugurated by Levinas.

The philosophy of Emmanuel Levinas also contributes to theory of language and signification, emerging as philosophy of language [59]. In Levinas’ philosophy of language, the expression “of language” is to be understood not as an object genitive, but as a subject genetive, which is to draw attention upon the idea of language that philosophizes, upon the propensity of language, the subject, to philosophize. This means to grasp that orientation which, according to Levinas, consists in the vocation of language for alterity, attention for the other. On Levinas’s account, this movement towards the other, “*autrui*,” is the a priori of language. It is in language that the relation between subject and identity is modelled. Therefore, at the basis of each identity, of all identities in which the I can recognize itself, there is the other, the relation of alterity. This is a central point in Levinas’s meditations: the alterity dimension at the very heart of identity (see Athanor, *La trappola mortale dell’identità* [11], Athanor, *Identità e alterità* [14]).

The expression “philosophy of language” *filosofia jazyka*, appears in the title of a book by Valentin N. Vološinov [120] (1895–1936), English translation *Marxism and the Philosophy of Language* (1973). Vološinov was one of the main members of the so-called “Bakhtin Circle” and the expression “philosophy of language” indicates the specific area of research. Bakhtin explains in “From Notes Made in 1970–71” (Bakhtin [21], pp. 349–374, Eng. trans. [20], pp. 132–158): on electing the expression “philosophy of language” rather than “semiotics,” the intention was to signal their critical standpoint towards certain trends in semiotics, those classifiable under such denominations in which these trends recognized themselves as “code semiotics” or “communicative (equal) exchange semiotics,” and again “code and message semiotics,” “decodification semiotics” [83]. Such trends were altogether distant from the approach adopted by Bakhtin and members of the Bakhtin Circle to the analysis of sign, language and meaning and certainly from tendencies that we associate today with studies by Charles Peirce and so-called “semiotics of interpretation” [28, 70, 71, 94, 95].

Mikhail Bakhtin and members of his Circle critiqued those trends in sign and language studies that reduce communication prevalently to a fact of transmitting intentional messages in the social, as though the message were a phenomenon that is already given and defined on the basis of a code, prefixed independently from the interpretive process. All on the contrary, when a question of live language, of the potential for sense and significance, messages and meanings are formed in the conmunicative process itself, they are in becoming, subject to multiple factors constituting the context of expression and communication. In the text there is the context; in the sign, in words, in the utterance there is the relation with the other, whether human or nonhuman.

Levinas’s reflections present an original contribution on the cultural scene of the twentieth century. From my own point of view I believe that associating the classics of semiotics in the tradition delineated by authors like those mentioned (of course there are others), representatives of so-called “semiotics of interpretation” and “semiotics of significance,” with “philosophy of language,” practiced according to the perspective described—all scholars and studies more or less from the same period—contributes to amplifying the originality of Levinas’s own approach, where *le trait d’union* with Levinas can be identified in the question of the relationship between transcendence and alterity as a determining factor in the development of sign, language and signifying process. They all somehow recognize alterity as a dimension that is essential to the sign, to communicating, interpreting and understanding. Consequently they each somehow elect as the object of their reflections all that which relates to the *centrifugal forces* in linguistic and social life by comparison to the *centripetal forces*. The scholars we have mentioned all work in one way or another on semantic gaps, on the *otherwise than being,* on the transcendent with respect to the ontological, on shifts in sense, on the processes of infinite deferral among signs in which the itineraries of sense and signification are constituted, therefore on differences and variations among signs in human life, individual and collective. Man is a sign, as Charles Peirce claims (see [101], see also Petrilli, “Man, Word and the Other,” in Thellefsen and Sorensen [119], pp. 5–12; Ponzio, “Not an Individual, but a Dual Self [at least],” in *ibid*., pp. 443–450), and the sign is in translation, it evolves and obtains in the relationship of dialogicality and alterity with other signs (Petrilli [73], Ponzio [103] and “Logic and Dialogic in Peirce’s Conception of Argumentation”, in Jappy [33], pp. 235–252).

## Dialogue and Alterity

At the origin of Levinas’s meditations, as anticipated, is logical-philosophical thought in the Greek tradition; and Levinas works on the Greek word in dialogue with the Jewish tradition and its writings. Europe, as Levinas says, is the Bible and the Greeks; and the Bible insists on responsibility for the other, even more, on love for the other. Justice could not find a better foundation. The implication is that for love of the unique single individual it is necessary to renounce the unique single individual; the demand is for the principle of equality. According to Levinas, Greek in the Occidental tradition is the sense of universality, it is the concept, abstraction, commnonality. In the Greek tradition the unique, the incomparable, the surplus with respect to a given identity must be reduced to the concept, the universal, to the community and its rules. As says Levinas in *À l’heure des nations* [53], humanity of the human must necessarily situate itself on the horizon of the Universal, learn from the Greeks, learn their word and their wisdom, Greek is the inevitable discourse of Europe, that the Bible itself recommends (It. tr., pp. 153–154). But to this tradition and the love of State justice the Bible adds its commandments demanding love, love without concupiscence, mercy and responsibility for the other, this is the other beyond the concept, the word, the law, my nieghbour. And for Levinas love implies care for the other, solicitude for the other’s well-being, charity for the other.

In his writings, Levinas dedicates special attention to the literary text. Under this aspect he shares with Mikhail Bakhtin, though independently of him, a love of Dostoevsky’s artwork. Like Bakhtin Levinas construes the concept of “exteriority”—Bakhtin speaks of “exotopy”, “extralocalization” (1920–1924, now in Bachtin e il suo Circolo [17], pp. 32–167, in English Bachtin [21])—on the viewpoint of literature, of literary writing. The analogy between Bakhtin and Levinas is extraordinary, as they both investigate the idea of mutually “finding oneself on the outside” in their description of the relationship between the I and the other. The importance of literary writing in Levinas’s philosophy, similarly to Bakhtin, is strongly attested in Levinas’s book of book of 1976, *Noms propres* [45]. But this dimension of his meditations is already traceable in his important essay of 1948, “La réalité et son ombre”:

On prend l’introspection pour le procédé fondamental du romancier […] Nous croyons au contraire qu’une vision extérieure—d’une extériorité totale […] où le sujet lui même est extérieur à soi—est la vrai vision du romancier. […] Même le romancier psychologue voit sa vie intérieure du dehors, non pas forcément per les yeux d’un autre, mais comme on participe à un rythme ou un rêve. Toute la puissance du roman contemporain, sa magie d’art, tient, peut-être, à cette façon de voir de l’extérieur l’intériorité, qui ne coincide nullement avec les procédés du behaviourisme (Levinas [37], 1982^2^, p. 114).

Bakhtin’s own dialogical thinking derives from his love for the multiplicity of different cultures, Occidental and Oriental, as much as from logical-rational traditions of thought and from religious thought in the Russian-Orthodox tradition. Thanks to all the languages it contains and in which it is determined, the viewpoint of Bakhtin’s own language, of his philosophy of language is strongly dialogized, polyphonic. Bakhtin’s characteristic dialogism is methodological as much as it is thematic: his thought is developed in dialogue among different sciences, disciplines and discourse fields, including the life sciences—biology, the hard sciences, chemistry, physics, but also music, etc.; and dialogism is also elected as the object of his reflections.

Though his “grand logic” was never published, with his pragmatism, or rather pragmaticism Charles S. Peirce too investigated total but not totalized experience in correlation with total and detotalized reality. Experience thus described is structured on the relation to alterity, he too presenting a vision that can be reconducted to dialogue among different cultures, traditions of thought, languages and disciplines including cosmology, psychology, phaneroscopy, metaphysics, theology, ethics and aesthetics, and literature with such writers as Edgar Allan Poe. Philosophical thought in Levinas as much as the exponents of the “major tradition” (thus denominated by Sebeok [114, 115]) in twentieth century semiotics as we are describing it, may be characterized as “religious” thought. Here, following in Victoria Welby’s footsteps, we use this term insofar as it recalls the Latin signifier “*religare*” (Petrilli [69], pp. 167–168, 586; Petrilli and Ponzio [96], p. 365). “Religious” therefore also in the etymological sense of uniting, relating, associating, putting together on a methodological level, beyond the specific content of research and beyond metaphysical-theological problematics.

Though they do not constitute our direct object of study in the present text, these authors too enter and influence our discourse, with Levinas. Beyond the topics thematized, the originality of Levinas’s engagement also emerges on a methodological level. The opening to what we might tag “material transcendence,” to semiotic and inferential infinity, to sense that is often elusive, ambiguous, paradoxical characterizes his discourse and under certain aspects associates him to semiotic studies in the tradition that focuses on the question of “infinite semiosis”, “reasonableness” before and beyond “reason”, and therefore on the question of “significance”. Beyond the letter, beyond the text and its contents, *alterity*: *specifically absolute alterity* by comparison to *relative alterity*. Alterity is revealed as the original (both in the sense of fundamental, essential and of primordial, first) dimension of the sign and of language. And with alterity just as fundamental is transcendence, signifying excess, surplus with respect to the said, to convention, to meaning, the law, to identity of whatever type, whether identity of the single individual or of a collective, group, genre, class, concept, etc.

To speak is inevitably to speak with the word of others, but it is also to demand listening by the other whatever the end of a given communicative interaction, even when a question of judging that other, of condemning that other to death, as Levinas says. To speak means to keep account of the other, in a relation of inevitable involvement, implication, responsibility such that to speak is always to respond, to answer, also in the sense of “to answer to” and “to answer for”—in the first place, to answer for oneself, to justify oneself (Levinas [56], Eng. trans. pp. 123–133, 144–148, 159–196, Petrilli [79]).

Identity articulated into social roles is differentiated on the basis of the alterity relation, but this is a question of “relative alterity,” a limited form of alterity, fixed within boundaries relatively to a given role and its responsibilities, necessary to the articulation of human behavior in the social. Instead, as observed, alterity that is not limited to roles and identities, that is irreducible to roles and identities, is something altogether different from relative alterity and from that type of sign relationship that Peirce denominates as “indexical”—the case of the relation between professor and student, father and son, wife and husband, in which one terms depends on the other and could not subsist without that other. Levinas calls this irreducible alterity “absolute alterity”. Peirce places this type of alterity in the semiotic category of “iconicity”, which tells of the capacity for sense on one’s own account (*CP* 8.332), Levinas’s *kath’autó*, the other in itself, without quiddity. In terms of Peirce’s phenomenology, absolute alterity is a matter of “firstness,” whereas “relative alterity” of the “indexical” type enters the category of “secondness” (*CP* 2.265, 2.283, 2.293–4, 2.305).

Unlike responsibility connected with role, with social position, that is to say, limited, special responsibility, responsibility with alibis, governed by identity logic, egocentric and indifferent to the other, responsibility concerning the absolute alterity of each one, unreplaceable singularity, is responsibility that cannot be delegated, that inheres to the relation of non-indifference to the other, of love and care for the other.

## Semiotic Materiality

The alterity relation is constituted as a relation with an excess external to the totality, as transcendence and proximity, contact with the other, encounter with the absolutely other that cannot be englobed, assimilated to identity, to knowledge, to the letter. Charles Peirce, Victoria Welby, Mikhail Bakhtin, Charles Morris, Emmanuel Levinas are all philosophers of alterity, excess, exotopy, dissymmetry; “philosophers of language” who “of language” valorize opening towards irreducible alterity, installment of a relation in which, to say it with Levinas from his book of 1961, *Totalité et infini,* “les termes s’absolvent de la relation—demeurent absolus dans la relation” [39]: 35–36. And through its paradoxical overtones, this statement succeeds in conveying the difficult ambiguity of human relationships, ultimately their essential poetic nature.

The relation to the other is one of in-compliancy, non-adjustment, inadequation, dissymmetry and non-assimilation in a relation to the other in its uniqueness, a relation that exceeds the limits of the known, a relation of non-compromission with Knowledge and Truth, of irreducibility to thematizing intentionality. With reference to philosophical-logical thought in the Occidental tradition, the effort is to account for the idea of infinity that inhabits us (Descartes), for knowledge capable of reasoning about the infinite in the finite, about that which the finitude of the *cogito* cannot contain, how absolute alterity is conceivable in the Same, about singularity, uniqueness that cannot be assimilated to the I (only the unique is absolutely other, as Levinas explains in *Entre nous* [56], Eng. trans., p. 174, 189–196; and uniqueness is the ethical experience of alterity, as Ponzio comments in *Con Emmanuel Levinas* [105], p. 262, remembering with Levinas that love is the condition of the very possibility of uniqueness, *ibid*., p. 168, 174–175), in the last analysis how in language there arises the desire of the infinite. In a book of 1982, *De Dieu qui vient à l’idée*, Levinas recounts how his conception of alterity developed beginning from *Totalité et infini*:

La tâche principale qui est derrière tous ces efforts, consiste a penser l’Autre dans-le-Même. Le *dans* ne signifie pas une assimilation: l’Autre dérange ou éveille le Même, l’Autre inquiète le Même, ou inspire le Même ou le Même désire l’Autre ou l’attend (le temps ne dure-t-il pas de cette pariente attente?). […] Le Même n’est pas, par conséquent, en repos, *l’identité du Même n’est pas à quoi se reduit tout sa signification.* Le Même contient plus qu’il ne peut pas contenir. ([47], p. 130).

It is in otherwise than being or beyond essence that language is realized in its materiality understood as irreducible alterity, what may be denominated “semiotic materiality” (Athanor, *Lavoro immateriale* [6, 70, 74, 75, 77, 89]). This is the material of signs understood as the capacity for resistance to manipulation, to englobement in the totality, excess with respect to com-prehension, to the order of discourse. “Material” is such with respect to an identity. The materiality, alterity, objectivity of signs emerges as such with respect to consciousness, the subject. But identity with respect to which the irreducibility of semiotic materiality, alterity, looms does not only concern the subject, but rather existence in its diversity, phenomena, events, concepts altogether different from each other. As emerges from the reflections of Levinas, the materiality of alterity regards such notions as freedom, commandmant, justice, responsibility, transcendence, but also the body, corporeality, work, war. Whatever the form of identity, it subsists as identity. The effective term of reference of the “material” is identity. Material is what cannot be reduced to identity. Material consists in alterity that resists identity (see Athanor, *Materia* [5], see [109]: 253–267).

To interpret the signs of the identity-alterity relation today is particularly important for the “general science of signs,” or “theory of signs,” or “doctrine of signs,” *semiotics*, above all when it is realized as *semioethics* (see also Athanor, *Semioetica e comunicazione globale* [12]). Semioethics works on the relationship between signs and values, semiotics and axiology, semiotics and pragmatics, as development of semiotics that precedes from John Locke and avails itself of subsequent contributions from scholars like Charles S. Peirce, Victoria Welby, from Mikhail Bakhtin and his Circle, and from Roman Jakobson, Charles Morris, Thomas Sebeok—who promoted the amplification of semiotics both on a theoretical level in terms of “global semiotics” and on a historiographic level reconnecting it to the semeiotics of Hippocrates and Galen—and, in Italy, Umberto Eco, Ferruccio Rossi-Landi, Massimo A. Bonfantini and Augusto Ponzio. Given the special bend of investigations on sign and language where the relationship between sign and value, language and value is central, the connection between such a tradition in semiotics studies and the work of Levinas is immediate. From our own viewpoint, in fact, another inevitable connection is that between “semioethics” and Levinas’s understanding of “ethics”: we have developed semioethics proceeding with Levinas, but also beyond in dialogue with the scholars mentioned and others still who in chronological terms came before him and after.

## Globalization and the Trap of Identity

Among the authors of contemporary philosophy, Emmanuel Levinas is doubtlessly the author who has most contributed to interrogating the Western vision of the world insofar as it is grounded in identity and monological egocentrism. Under this aspect too his writings can be related to contemporary semiotics reoriented in the direction of *semioethics*.

Levinas has insisted particularly on the need of refounding and reformulating humanism, on the possibility of a *new humanism* outside the traps of anthropocentrism and egocentrism, a new humanism which he indicates as the “humanism of the other man” (an expression which gives the title to his book of 1972, *Humanisme de l’autre homme*), a centrally important concept in the architectonics of his thought system. Levinas’s new humanism is founded on the other, emerging as “humanism of alterity” by contrast with traditional “humanism of identity”. Alterity implies the capacity of openness to the other, an orientation that contrasts with Western thought when it legitimates the reasons of identity and predominates over the other, to the very point of recognizing the reason of war. Levinas elaborates on the problem of war, projected as the truth of reality, in his Preface to *Totality and Infinity* (pp. 21–30). War is the ugly face of the real, of real politics. War suspends moral imperatives and praises politics as the art of winning by all means possible. The logic of war is the realistic logic of being, of identity, of ontology and politics.

But the human subject cannot be reduced to the logic of identity and the social roles through which it is articulated. Humanity transcends and at once subtends the logic of roles and identities. This is a question of the *absolute alterity* which resists all attempts at englobement on behalf of closed, totalizing, identity logic, contrary to *relative alterity*, a limited form of alterity necessary to the delimination of roles and relative responsibilities.

By comparison with dominant centripetal and egocentric tendencies, the humanism of alterity, absolute alterity, privileges the centrifugal movement of encounter with the other, of responsibility for the other to the very point of placing the existence of others before my own. The original relation to the other is characterized by “non-indifference” towards the other, by responsibility for others, by love and charity for the other, by the impossibility of avoiding implication in the life of others. To proceed with Levinas is to recover this relationship of non-indifference, involvement, compromission in the face of the experience of others (see “Humanisme et an-archie,” in [43], pp. 71–93). On the background of such a philosophical horizon, semiotics, “global semiotics,” oriented in this direction assumes a perspective of the ethical order.

The question of identity-alterity is centrally important today in the reality of globalization and inevitable encounter among cultures (see Athanor, *Globalizzazione e infunzionalità* [10]). Dominant ideology connected with the current economico-social order is characterized by the tendency to homologation as imposed by equal exchange market logic (Rossi-Landi, Schaff, Vaughan). Global communication-production, the expression of advanced capitalism, always ready to invest in “human capital,” is noteworthy for its “destructive character” (Walter Benjamin): destructiveness has now reached planetary dimensions, as evidenced on a political level by facile recourse to war and it logic—military interventions of various sorts, military meddling, so-called “humanitarian wars” (*contradictio in terminis*, as though there could exist such a thing!) (v. Athanor*, Mondo di Guerra* [7], Athanor, *Umano troppo disumano* [9, 104, 108]). Homologation and destruction are inevitably accompanied by the opposite tendency, the need to assert identity and belonging (in the sense of blind affiliation to an identity), by the defence and exportation of values reflecting “our lifestyle,” the “ascent of subjectivity,” demands and expectations related to blind identity, that is identity indifferent to the the other, self-confidence projected in terms of self-exhaltation, security for self alone. All this goes hand in hand with the arrogance of reason, the exhaltation of technique, productivity, exploitation of work, relentless reduction of the social to a work community, a community of “workers,” now that the inexorable perspective is a society of workers without work (cf. [1, 110]). The presentday globalization system is destructive not only of products, of the instruments of work now become “intelligent machines,” of “jobs,” but also of the natural environment, the body of each one, of the quality of life made to depend on indifferent work, reduced to alternation between work-time and freetime, or emptied and impoverished by the lack of work, by unemployment (see Athanor, *Semioetica e comunicazione globale* [12]). In this context, identity, understood as difference, finds it ever more difficult to assert itself, thereby giving rise to a paroxystic search for identity itself. But as Charles Morris prognosticated in his book of 1948, *The Open Self* (the Italian edition, in my translation, bears the subtitle *Il soggetto e le sue metamorfosi*), this is a question of closed identity, identity ready to sacrifice the other and that does not know, even less recognize, alterity as the very condition of identity, as Levinas would say, of its constitution as sign, as Peirce teaches, in whatever form, including the human being as a sign.

War presents the most conspicuous face of today’s globalized social production system in its most destructive aspect. This system which we have charatacterized as the communication-production system, is also the *communication-production of the war system* (Petrili and Ponzio [92]). The war industry in interested in new markets and in obtaining consensus for war, for the recognition of war as *just* and *necessary*, a legitimate means of defence against the menacing “other,” and a means through which to impose the “rights of identity,” the rights of “one’s own difference”. This is what emerges clearly from relatively recent events in our globalized world, and above all for what concerns the solution to international conflicts and strategies adopted to counteract Islamic terrorism. But the point is that it is not the “other” *per sé* who threatens or destroys identity and difference, but this very social system that promotes determinate identities and determinate differences, even paroxystically, to the point of self-exhaltation, exhaltation of identity, identity closed to the other in whatever form.

Claims are made to one’s own rights, passed off as human rights *tout court*, and in the name of human rights and social justice the request of hospitality on behalf of some extracommunitarian, of the person without “citizenship” is rejected. Reference here is to citizenship with respect to some identity, with respect to affiliation to a group of some sort: not only the migrant, the refugee, the non-white person, the homosexual, transgender, mentally ill, but also the woman, child, the foreigner who speaks a different language from my own, the person who professes a different religion, the poor, those excluded from the circuits of technological communication, from the privileges of so-called developed world (cf. [87, 88, 90]). Here “human rights” are substantially the rights of the I, of Westernm globalized identity, reduced to the status of consumer, motor of the global market.

## Human Rights and Rights of the Other: For a New Humanism

The question is how to manage the current situation, how to get free of subservience to identity functional to reproduction of the communication-production system, to identity as pervasive as the very system, itself pervasive both in quantitative terms, given its extension over the entire planet, and in qualitative terms given that it invests life in all its aspects. With Levinas and the exponents of twentieth century semiotics mentioned, the beginning of an answer to the question is traceable in the concept of *semiotic material*, that is, *material as alterity*, where by “material” is understood that which exists *per sé*, on its own account, *kath’autò*. The allusion here is not to relative alterity, but to absolute alterity, constitutive of identity itself. The way out is a *new humanism*, *humanism open to alterity*, *humanism of alterity*; with Levinas: “humanism of the other man”; with Morris: humanism of the “open self” and therefore encounter among alterities, singularities, among identities in their uniqueness. Beyond identity and its closures, beyond the processes of identification, affiliation, empathy, rejection—on the political-national level, on a broadly cultural level, on a logical, psychological and emotional level, the material of alterity preexists and resists at varying degress of alterity and dialogicality, to the very point of presenting a degree of irreducible alterity, “material” precisely, finding expression in some “sign residue,” to evoke Ferruccio Rossi-Landi’s terminology (see [111–113]), surplus, significance, final interpretant [62], obtuse sense [23]. No doubt the materiality of one’s body also makes itself felt: my body gets tired, sick and dies just when I wanted it to start a new business, finish an essay, care for the children. And respect to the otherness of one’s own body, the alterity of “others,” of the other as other (*autrui*) is even more material, even less manageable, less manipulable, less reducible to the demands, needs, will of identity, as despotic as it may be. The person who resists, who won’t surrender, who persists in one’s alterity, who insists on asserting its own alterity can certainly be eliminated, killed, executed: but this is to declare the failure of identity, of the reason of identity, of its argumentations, of the claim to operating in the name of justice. Alterity that resists on the cognitive level, on the evaluative level, on the level of practice and planning, alterity that identity suffers in spite of itself, is alterity inherent to very identity, the infinite in the finite, absence in presence, the invisible in the visible, *per invisibilia visibilia* (see [68]), alterity perceived from within the very same processes of identification—as Peirce says, at the heart of the sign’s identity there is alterity (see [73]).

“Les droits de l’homme et les droits d’autrui” (Human rights and rights of the others) is the title of an essay by Levinas if 1985, originally published in the collection *L’invisibilité des droits de l’homme* (now in Levinas, *Hors sujet*, [52, 53]). In the form of a paradox this titles evidences how the *rights of the other* may not necessarily enter *human rights*, in other words that *human rights* do not necessarily foresee the *rights of alterity*, that human rights today and ever more are the rights of identity, of the “closed self,” while the rights of the other are denied. Thus understood so-called “human rights” consist in excluding the rights of others: and this is clearly visible where responsibility for the other is neglected (cf. Athanor, *Diritti umani e diritti altrui* [15]). A contradiction emerges here, which is not simply of the logical order, between the claim to human rights and what is understood by “*droits d’autrui*” (Levinas), being a contradiction that arises from identifying human rights with *the rights of identity*, of the Occidental subject and lifestyle (as recites the White House document released in 2002—a year after the attack on the Twin Towers—which introduced the idea of “preventive war,” to counteract the terrorist politics of “Rogue Sates,” (cfr. [31, 32]). In this world made of walls and barriers, human rights are the rights of belonging, affiliation, of the privileged community, closed and exclusive.

Humanism that in terms of etymology attaches to *Homo* remains as humanism of belonging, of identity, and as such it implies the processes of identification and with identification the arbitration of esclusion. With Levinas and with the masters of the sign that we here remember, a new conception of humanism is perspected: not as deriving from *homo*, but from *humanitas*, which, like *humilitas (humility)*, derives from *humus,* humid mother earth cultivated together, a humanism of alterity. This is the humanism that—as we have already mentioned—Levinas [43] denominates “humanism of the other man”. With this conception of humanism there at last vanishes the illusion that we may trace the possibility of escaping conflictuality—which all partial totalities involve and all identities that appeal to them—in the broader, more general, universal category, that is, *Homo*, humankind. In this case there will always be somebody who is less human, inhuman, and someone who is more human than others, just as in the George Orwells’s *Animal Farm*, among the “equal”, there was always somebody “more equal than others”.

As inscribed in semiotic material, the material of alterity, human rights derive from an original relationship with the other, preceding all legislation and all justification. In this sense they defer to a relationship of unindifference, of involvement, of responsibility with the other and for the other, that could be described as a priori with respect to the “declaration of human rights,” insofar as they are antecedent and independent from roles, functions, merits and recognitions. Human rights, but as *including and non excluding the right of the other*, are a priori with respect to all permisssion, concession, authority, demand of one’s own rights, a priori with respect to all tradition, jurisprudence, privilege, belonging, affiliation, before all reason. Human rights cannot not be also the rights of others, and therefore effectively human rights, the rights of *humanitas*, the rights of the common care of mother earth, that already in terms of etymnology recall the concept of *humilitas.*

## Ethics

In Levinas’s philosophical meditations on language there resounds the relationship to ethics, but this is not ethics as traditionally understood. Ethics in Levinas’s works does not resound in the sense of morals, as a branch of philosophy, a program or decalogue intended to regulate human behaviour. Levinas does not consider it his task to build an ethics, but to search for its sense. As he declared himself in his interview of 1981 with Philippe Némo, he was not interested in ethics but in what makes ethics possible (*Éthique et infini*, 1982, tr. it., p. 105). Ethics for Levinas is “first philosophy” (*Totalité et infini* [39], tr. it., p. 313). Transcendence is ethics (*Dieu, la mort et le temps* [56], tr. it., p. 295).

In Levins the terms “ethics” and “ethical” connote what in language is an excess with respect to the cognitive sphere, thematization, ontology. They testify to the condition of “proximity”, “responsibility” and “substitution”, of “involvement”, “co-implication,” “intrigue” with the other as a characteristic of alterity. Under this aspect, particularly significant is Levinas’s essay of 1968, “La substitution” (now in *Autrement qu’être ou au-delà de l’essence* [43]) and “Language et proximité,” of 1967 (published in the second edition of his *En decouvrant l’existence chez Husserl et Heidegger*, [37]): though published separately, these essays are united in the Levinasian conception of “otherwise than *being*,” of “beyond essence!”.

The special sense according to which the terms “ethical” and “ethics” are understood by Levinas is described in a note to his essay “Langage et proximité” (p. 225):

We call ethical a relationship between terms such as are tied neither by a synthesis of the understanding nor by a relationship between subject and object, and yet where the one weighs or concerns or is meaningful to the other. (Levinas [38], 1967 edition, Eng. trans. [53], p. 116)

The relation to the “other in one’s otherness” constitutes the foundation of ethics, in other words, this relation comes to fruition as ethics. With Levinas, therefore, the terms of the “ethical” relation are neither connected by a synthesis of understanding, nor by a relation between subject and object. And yet in this relation one term counts for the other, has value for the other, concerns the other, is significant for the other. The relation to the “other in one’s otherness,” “in one’s alterity” is a relation which can neither be exhausted nor unraveled by knowledge, the concept, abstract thought. Ethics, according to Levinas, comes before and after ontology, before and after the State, before and after politics, given the implications of ontology in political realism, but it is also “ethics beyond ethics” [30, 31]. As stated, this constitutes an approach that is altogether different from “ethics” as it is traditionally understood. Levinas thematizes “ethics as first philosophy” in response to the problem of the relation to the other, to the other’s singularity as other, a relation charged with responsibility towards the other, irreducible to a moral formula, to moralistic certainty and to self-righteous assurance.

Levinas clarifies that by “first philosophy” he intends philosophy of dialogue, therefore philosophy that cannot not be an ethics. Ethics for Levinas implies dialogical encounter with the other, it manifests itself in alterity, in the primordial “for-the-other”: “even the philosophy that questions the meaning of being does so on the basis of the encounter with the other”, as says Levinas in “La proximité de 1′autre” (1995[1986], Eng. trans. p. 98). The origin of meaning, of intelligibility, of rationality is in the face of the other, in responsibility for the other man, so that ontology, objective knowledge, politics have meaning thus conceived at their foundation. They are “commanded” by meaning as responsibility for the other, by unrest for the other prior to representation of the other in the social, in the community.

The plurality of human beings with whom we are necessarily implicated makes justice necessary and with justice the objectivity of knowledge, fairness, the impartiality of judgement; the multitude involves the need to set up law courts and political institutions and, paradoxically, even a certain violence, in fact implied in all justice: to defend the other, my neighbour, as Levinas observes in “Violence du visage,” always involves a certain violence for someone (1995[1985], Eng. trans. p. 172).

That ethics is “first philosophy” means that ethics, as described above, comes before ontology. Levinas repeats this: reason, knowledge, ontological thought are founded on ethics and must be nurtured by ethics, inspired by ethics. At the basis of all forms of humanism in sociality there is the for-the-other, attention for the other, thinking for the other, preoccupation, unrest for the other. This is the original condition described by Levinas in his book 1972, *Humanisme de l’autre homme*. The condition of good and evil, of their possible existence is the *other*. There can be neither goodness nor evil, neither cruelty nor justice nor injustice without reference to the other. Both good and evil as characterizations of human doing presuppose alterity, addressing the other, they depend on the existence of others. In semiotic, or better biosemiotic terms, alterity is what moves the advancing of semiosis and therefore of life, the orientation of communication and, in the specifically human world, the intentionality of non-verbal behaviour and of the word in “good” and in “evil”. All encounter, says Levinas in “La proximité de 1′autre,” begins with a benediction, contained in the word “hello,” which is already presupposed by all *cogito*, all reflection. Addressed to the other man this greeting is an invocation: “I therefore insist on the primacy of the well-intentioned relation toward the other. Even when there may be ill will on the other’s part, the attention, the receiving of the other, like his recognition, mark the priority of good in relation to evil” (Levinas 1995[1986], Eng. trans., p. 98).

This is the meaning of the term “ethics,” that is, ethics at first philosophy, that we too reading Levinas, but also Peirce and Sebeok, have attributed to the term “semioethics” (cf. [70, 74, 93, 96, 97, 109], pp. 11–12).

Considered in such a framework, subjectivity, identity of the each one, develops and finds expression in the relation of alterity among signs not only within human cultures and societies, but also in the great sign network that is life over the planet, following a line of development in semiotics which beginning from Charles Morris finds its explicit characterization with Thomas Sebeok as “global semiotics”. From this point of view, that of global semiotics, semioethics can also be considered as having an indispensible, unavoidable vocation of the ecological order.

The question of identity as traditionally conceived by Western thought, that privileges the theoretical, representation, knowledge, the subject-object relationship, finds its most rigorous interrogation in Levinas. His starting point is the I-other relationship considered in terms of involvement and responsibility, of relation, as he says, of “one-for-the other,” to the point of “substitution,” of even becoming the other’s “hostage” (Levinas [44], Eng. trans. pp. 113–118). In fact, in Levinas, as we have already observed, the I-other relationship, the other from self and the other of self, is one that cannot be explicated uniquely in cognitive terms. I and other are not bonded in a theoretical synthesis, nor is this relation reducible to the subject-object relation. Rather than resulting from a process of abstraction, the I-other relation is *a relation of involvement among singularities*, founded in the body, whose distinctive characteristic is alterity precisely, non-relative, absolute alterity.

Levinas writes “Other” with a capital letter, with which he intended to specify that his reference is not the other as this other is interpreted, evaluated, understood, tolerated, rejected, and in any case rendered an object. Instead, this is a question of the other on its own account, the other as a value in itself, as absolute alterity. The I-other relation is a relation among differences, alterity-differences, among singularities non-indifferent to each other, a relation of participation and responsiveness among unique, unrepeatable terms that together constitute an open community, in relations of intercorporeity and thus of dialogic interconnection. As such the I-other relation precedes all initiative taken by the I, all concession from the I, all opening by the I, and instead constitutes the very condition and “material,” the “sign material” of the I. This description of the I-other relationship is also amply illustrated in Peirce’s semiotics.

## The World

Reading Levinas the limits of the “World” are the limits of the World as-it-is, in its identity. The World thus described is associated with naïve and dogmatic forms of realism, a world defined in terms of ontology, of being this or that, of Being. This World is the result of dominant ideology and is established by the Order of discourse [36]. The subject that inhabits the World thus described aspires to coherence, to security, it engages in its own *conatus essendi* and tends exclusively to its own self-preservation. Such a subject is characterized by a “clean conscience,” declaring to be at peace with its conscience in conformity with the rhetoric of political systems and mass-media. All this is functional to homologation in a totalizing world, to dominant ideology, the “ideo-logic” of a globalized world. And yet that special semioses, different cultures and languages, historical-natural languages, special languages within the single language flourish is indicative of the human potential for resistance with respect to the “trap of identity,” in front of the reduction of values to the dominant values of the “equal exchange” market, of giving for the sake of receiving, of power and control over the other.

The question raised by Levinas is whether there be no other sense than that of being in the World and for the World; whether the *properly human* can exceed the space–time of objects, the space–time of identity, of “closed identity,” as denominated by Charles Morris in *open self* [60], whether there exist relations not reducible to identity conceived in such terms, that evade the relation between subject and object, the relation of exchange, equivalence, functionality, self-interest, productivity; whether there exist interhuman relations *altogether other*, and yet at once material, earthly, intercorporeal (Levinas [56], Eng. trans., pp. 91–102),whether there exists *another sense*, *a sense that is other* with respect to the sense of the World as-it-is, the world of objects and relations to objects. Such questions are oriented in the direction of a *new humanism*, a humanism other than the “humanism of identity”, that is, the *humanism of alterity* (cf. [72]).

For Levinas the “properly human”—where alterity is not at all abstract but has a “material” valency,” such that it counts above all in terms of *intercorporeity*—consists in the “superfluous,” in the “nonfunctional”, beyond need, in *desire*. The ideology of functionality, of productivity, of competition is sustained by social roles based on egocentric identity, on self-interest, which ordinarily orient individuals in respect of their roles. The properly human, instead, consists in a “movement” without return, also in the sense of “without profits,” in an exit from self, also in the sense of beyond one’s own time, the time of one’s own life, beyond contemporaneity, towards that which is other, otherwise than being, “being like this”. Levinas calls this movement *œuvre* and indicates the place of its realization firstly in the artwork (see “Le sens et l’œuvre,” in *Humanisme de l’autre homme* [42], pp. 42–47). This movement is without return to the “subject,” it implies exposition—at a risk—to alterity, hybridization of identity, fragmentation of monologism, evasion from the subject-object relation. All properly human products, all properly human actions have the characteristics of the artwork. It is not incidental that in human expression, human action, in human artifact, human products there is generally something extra, an excess with respect to function, an element of non-functionality (cf. [107]): this is what characterizes the human in its specificity as human, a sort of “signature” indicating how that which has been produced is the result of the hand of the human, of humankind. This movement cannot be englobed by the logic of roles, by the logic of belonging to identities, aggregations and assemblages, but on the contrary transcends and at once precedes such logic.

## Outside Subject

*Hors-sujet* is the title of a book by Levinas [52, 53]; “hors-sujet”, outside subject, also off the subject, in the sense of not responding to thematization, representation, outside the ontological cages of being. This orientation is founded on alterity. A condition of this *new humanism*, the humanism of alterity, is interrogation of the “clean conscience,” the “conscience at peace”. Such a philosophical orientation inevitably raises the need for a reconsideration of “human rights” in light of the rights of the other, by contrast with their common interpretation in terms of the rights of the self, of identity (see “Les droits de l’homme et les droits d’autrui”, in Levinas, *Hors sujet* [52, 53]).

The places that best evidence the properly human are the places where one’s own time encounters the time of others, not “work time,” but “unproductive time,” “disposable time,” the time of alterity, one’s own alterity and the alterity of others. This is the time of commitment, dedication, the welcome, hospitality, the time of friendship and love, the time of the maternal and of care, of mothering and nurturing, the time of inventiveness, of musement, the imagination, the ephemeral, the ineffable, the time of aesthetic activity (of literature, the figurative arts, music). This is the time of excess with respect to closed identities, with respect to the finite time of the life of each one, situated between birth and death, this is the time of dialogical de-totalization and of the manifestation of differences beyond their oppositions, beyond their integrations, beyond their recruitment at the service of the World as-it-is. Although the current social system is based on self-interest, gain, profit, and is a function of the egoistic, egocentric self, we know that care for the other, the maternal, friendship, love are also part of the system, which means that relationships and feelings that regard each one of us in our total non-functionality, that involve us as an end and not as a means, are also included (see Levinas, *Dieu, la mort et le temps* [56]).

## Responsibility and Substitution

In a conversation between Emmanuel Levinas and Augusto Ponzio, which took place on 20th November 1988, at the home of Levinas in Paris, rue Michel-Ange, the French philosopher from Lithuania explains his conception of “substitution”. At the time, in addition to numerous essays, Levinas had published the important monographs *Totalité et infini*, in 1961, *Humanisme de l’autre homme*, in 1972, *Autrement qu’être ou au-delà de l’essence*, in 1974, and finally *Hors Sujet*, in 1987. Ponzio is the author of the first monograph on Levinas worldwide, *La relazione interpersonale*, published in 1967, followed by his French monograph, *Sujet et alterité. Sur Emmanuel Levinas* (l’Harmattan), in 1996.

Levinas associates the notion of “substitution” to “responsibility”. “Responsabilité et substitution” is the title of the 1988 conversation just mentioned, published in *Sujet et alterité*, by Augusto Ponzio (now in *Con Emmanuel Levinas* [105], see also Ponzio [105]). To substitute the other does not mean to identify with the other, instead, each one remains in one’s situation of incomparability, even on handing oneself over as “hostage” to save the life of the another. But apart from extreme situations of this sort, the concept of substitution according to Levinas distinguishes itself clearly from situations indicated with the name of empathy, sharing, participation, *einfülung* where convergence occurs between two distinct identities to the point that where there were two, therefore where a relationship presents irreducible otherness, now there is one only. To substitute the other implies to “bring comfort” to the other, to take responsibility for the other, to take the burden upon oneself of the other’s needs and preoccupations (see “La substitution,” in Levinas, *Autrement qu’être ou au-delà de l’essence* [43], pp. 156–205).

The other is a constitutive impediment to the integrity and closure of the word and of consciousness as identity, totality, being. The relation to the “other in its alterity” presents itself as a relation of excess, as surplus, as the capacity to transcend objectifying thought and the relative constitution of objects and concepts. The relation to the other as other, the relation of alterity, irreducibly transgresses the sphere of knowledge, of the concept, of abstract thought, although it at once forms the basis of the latter, as its condition of possibility. The relation to the “other as other,” to the “other in its alterity” is involvement, exposition, responsibility. Here *proximity* loses its obvious meaning of spatial vicinity to resound in an ethical sense and say of the inevitable implication of one’s own life in the life of the other: *my neighbour* is him for whom I am responsible, with whom, at each occurrence, I am inextricably involved.

## The Face

Levinas characterizes the I-other relation as a relation with the “face” of the other, as a “face-to-face” relation (see Levinas, *Totalité et infini. Essai sur l’extériorité* [40]). This latter expression does not imply that the relationship to the other must occur at the level of physical encounter. The language with which the other manifests itself is not only oral language. The face of the other also finds expression in “writing” (see below Sect. 11). “Face” for Levinas has a metaphorical signifying function, it signs alterity outside habits, masks, outside the relation among “persons”. Phenomenology of the face: the face tells of the relation of alterity, which is not thematization of knowledge, but an exteriority; the face tells of the I, in front of the face, outside subject (“Le visage et l’exteriorité,” *Ibid*., pp. 203–277). To the word “face” Levinas generally adds “nude”. The nudity of the face is absolute alterity in the I-other relationship. Therefore, in Levinas the idea of opposition between speech and writing—which certain critics have mistakenly wanted to attribute to him, between spoken language and written language no longer holds, it is no longer implied. Well before it is fixed in representation, the face is already language, it presents itself as interrogation in front of the I, as an appeal to the responsibility of my being in the relation to others. In front of the face of the other, the I is interrogated. Through its nudity, exposition, fragility, the face says that its alterity will never be eliminated. The alterity of the other resists to the point that, in the attempt at eliminating the other, it will be necessary to resort to homicide and war—which constitute evidence of the other’s irreducibility and of the I’s failure to prevail over the other, to overcome the other, to get to grips with the other, if not through violence.

The Other, *Autrui*, as Levinas says, puts the I into the accusative, summoning it, questioning it, calling it back to the condition of absolute responsibility, responsibility of the I as other with respect to all other identities. This absolute responsibility is responsibility for the other, responsibility understood as answering *to* the other and as answering *for* this same other. I must answer to the person I am called to answer for: responsibility in the face of the person I am responsible for, responsible for a face that re-gards me.

This is a type of responsibility—unlimited responsibility, out of role (“moral responsibility,” as denominated by Mikhail Bakhtin, by contrast with limited, defined, “technical responsibility”, “formal,” “special” responsibility) –, unlimited responsibility that does not allow for rest and peace, which instead responsibility with alibis, limited responsibility intends to ensure: in the light of absolute responsibility, the peace of a “clean conscience,” peace as a truce, peace functional to conflict, to war, reveal themselves in their misery, in their inconsistency, in their insubstantiality.

We are outside the sphere of “interest,” “self-interest,” outside those forms of the interpersonal relationship—including not only exchange relations, but also relations of “friendship,” “love” (on this account there exist such expressions as the Italian, “amicizia interessata,” literally “self-interested friendship,” that is, friendship based on egoistic self-interest, one-sided friendship)—based on a precise end, a return, a gain, on a profit of some sort. Levinas uses the expression “*dés-inter-essement”* to indicate a movement towards the other which is “otherwise” with respect to all this (see “L’argument,” in Levinas, *Autrement qu’être*, [43], pp. 13–39).

In front of the other persistence in one’s own being is suspended (see “La persistenza dell’alterità” [109], pp. 17–20). Even the same *conatus essendi* takes second place. And no other can assume my responsibility. Each I finds itself in the condition of irreplaceability, and it is precisely this condition that renders each one, each I, unique. I and other in their uniqueness, in their singularity are not simply individuals of a genre, of an assemblage, a group, a class, anonymous members in the logical extension of a concept. To this uniqueness of the I there corresponds the uniqueness of the other, the face of the other as other. The impossibility of evasion from responsibility, of withdrawing from responsibility, does not imply a relation of passive dependency. Levinas speaks of this relation in terms of “election,” of “elevation”. In the one-to-one relationship between unique single individuals is achieved the I’s “freedom,” the possibility of deliberation: it is the other who, with the face that gazes at the I, “re-gards” the I, placing the I in front of an interrogation, a request, a choice, “orders” the I, “elevates” the I to the condition of freedom, renders the I conscious of being free. The vocation for Being (Heidegger) is interrupted by another vocation, that for the life of the other, for the other’s destiny, such that I am immediately responsible for this other in his or her daily trials.

In the Western tradition closed identity has generally prevailed over non-indifference; relationships among individuals belonging to the same genre, to the same class, the same group, bonded by the same community identity, with ever more restricted responsibilities, have always prevailed; the logic of identity indifferent to relationships without alibis, among singularities, has always prevailed.

The capitalist world has constructed its socio-economic reproduction system on identity, bringing it to the point of exasperation. Capitalist ideology has even exploited the subject’s fear of the other—the other object of fear experienced by the subject—to paroxystic degrees, ever more attenuating and transforming the propensity for fear *for* the other, into fear *of* the other.

## Fear of the Other

Fear *of* the other, the fear I experience in front of the other, is closely connected with the constitution of identity. Whether individual or collective identity, the institution of identity requires separation from the other, delimitation of interests, on the basis of which is determined that which belongs to identity and that which does not belong, that which regards identity and that which does not regard it—as much as the gaze of the other, however, regards me always (both in the sense that it concerns me and in the sense that it gazes at me, looks at me, watches over me, from the French verb *regarder*). Identity determines and delimits, demarcates responsibility. Identity is delineated on the basis of difference, identity-difference, but difference and identity call for indifference. Difference understood as identity relates to a given genre, class, community and requires indifference to the other, lack of interest in the other, disinterest, disregard towards the other, absence of fear *for* the other. Difference and identity call for circumscribed and limited responsibility which begins and ends in a genre, a class, an assemblage of some sort, with the function of guaranteeing identity. *From non-indifference to the other to difference and relative indifference*: this is the trajectory through which identity is constituted and delineated.

Closed in our identity, what which concerns us is progressively reduced to that which regards the interests of identity, a reduction that finds its justification in limited responsibility sustained by alibis. But the more we forsake *fear for the other*, the more *fear of the other* increases, to the point of exasperation. Here “fear *of* the other” is the fear that the other provokes in the subject, the other object of fear: therefore “of the other” is understood as an object genitive. But “fear of the other” can also be the fear that the other perceives. In this case “of the other” is a subject genitive. Logic distinguishes between the *subject genitive* and the *object genitive*. If in the expression “fear of the other”, “of the other” is understood as an object genitive, and not as a subject genitive, then it is the other, the object, whom the subject fears, and it is not the other who fears, it is not the other to be the subject of fear, it is not the other to experience fear. But there is a third case that logical analysis does not generally foresee: to feel the fear of the other as *feeling the fear that the other feels, to feel the other’s fear*, to perceive the other’s same fear, *therefore to fear for the other*, to be concerned for the other, to feel “fear *for* the other,” precisely. Here the distinction between subject and object no longer holds. With Augusto Ponzio ([109]: 115), we may denominate this third case of the sense of “fear of the other,” the “other’s fear,” thus “fear for the other,” the *ethical genitive*.

In today’s world the expression “fear of the other” has two main meanings. It either indicates that the other is the cause of fear, or that the other is afraid; that I fear the other or that the other fears me. That which is ever more circumscribed, generally circumscribed to the “private” sphere, and that nonetheless is still perceived and cannot be extinguished, is fear of the other perceived as fear *for* that other. But this is exactly the type of fear that is urgent to recover, and to recover within the “public” sphere, and not only in the “private” sphere: fear *of the other*, where “of the other” is not a subject genitive (the other who fears), nor an object genitive (the other who provokes fear, who is the cause), but a sort of *ethical genitive* (the other’s fear), therefore fear for the other, fear for that other’s life conditions, precariousness, difficulties. This case does not need to be invented in logic because, as Levinas says, it is the first case in the relation of alterity. Instead, the point is to recover the ethical genitive at the very earliest and to put it first. Fear *for the other* is the condition for preventive peace, which Levinas calls “*primordial*”.

The essential characteristic of social relations in the world today is that they present themselves as relations among individuals who are ever more indifferent to each other, ever more isolated from each other. To this characteristic is connected separation between public behaviour and private behaviour in the same individual subject, therefore also indifference among roles, competences, tasks, languages, responsibilities, even within one and the same individual, the same subject, as the standard modality of undersigning and conforming to the social system one belongs to, is affiliated with.

A paradox connected with globalization today in its current phase of development is that social relations emerge as relations among individuals who are isolated with respect to each other, who are mutually indifferent to each other. The relation to the other is suffered as a necessity to the end of achieving one’s own private interests, egocentric self-interests. And exclusive preoccupation with one’s own identity, with one’s own difference indifferent to the difference of the other, enhances fear of the other, in the sense of fearing the other. So then, in the modern world, fear of the other, fear that the subject experiences towards the object, reaches paroxysmic degrees. But, contrary to Thomas Hobbes’ conception, the situation of *homo homini lupus* is the point of arrival in the constitution of identity and not at all the point of departure (Petrilli and Ponzio [96]: 41–44).

## Alterity and Justice

The rights and freedom of the I are instituted in front of the need to answer to the other, under the weight of unlimited, absolute responsibility for the other. The I is originally exposed to interrogation by the other and such interrogation is at once constitutive of the I and its freedom, insofar as it sanctions the transition from spontaneity to consciousness, from freedom as passive *jouissance* and happy spontaneity of the I, to freedom as a right and speaking that right. The origin of the I, an origin without *arché*, in this sense *an-archical*, is in the uncomfortable conscience, a conscience ill at ease, a bad conscience in front of others, in the need to justify one’s own presence, in one’s own responsibility without alibis and without the possibility of evasion from the other. From the very beginning, the I’s conscious is ill at ease (see “La conscience non intentionnelle,” 1983, in Levinas, *Entre nous. Essai sur le penser-à-l’autre* [55], Eng. trans. pp. 123–132). The I in the nominative, the I understood as subject, as intentional consciousness, as one’s own word, derives from interrogation of the I, from the I put into the accusative, from the continuous effort to achieve a comfortable conscious, a conscious at ease, a clean conscience, a conscience in peace. The I that must strive to answer for itself, transforms into the I as it normally presents itself, as the I in the nominative, as subject, capable of making decisions and self-sufficient. From interrogation there also derives the freedom of the I, there also derive the rights of the I, “human rights”—elaborated to defend the I in front of the face of the other, the I summoned by the face of the other, to account for the rights of others, in this case to defend itself as “I”.

A just State must be established with just laws in order to guarantee freedom and avoid the danger of tyranny (Levinas [40], pp. 334–336). Order based on the logic of closed identity, of difference indifferent to differences can have a boomerang effect on the I, can backfire on the I in the form of fixed and inflexible laws, these too tyrannical and violent. Law thus conceived is based on the rights of the I, regulated by closed identity—in the extreme form even to the point of commanding war, considered as the inevitable means of defence, the realistic face of being, of the self-interests of the individual and of the community. The I is open to blackmail by the impersonal order, to the point of having to accept, in the name of freedom, its own, the *extrema ratio* of war. The fallacious reasoning at the foundations is that violence can only be stopped through violence, through suppression. The being of things administered realistically by the impersonal discourse of law—in the context of which war is presented as ineluctable violence and as self-sacrifice—has its *otherwise than being* in its very foundation, in the face-to-face condition.

This condition is even more realistic, truly realistic: does not the face-to-face condition, the relationship between one will and another will, as Levinas says, imply “a relationship of command without tyranny, which is not yet an obedience to an impersonal law, but is the indispensable condition for the institution of such a law?” (Levinas, “Liberté et commandement” [38], Eng. trans., p. 18). The opposition of a nude face, the opposition of disarmed eyes, with no protection, beginning from which the I is constituted as responsibility, is not the opposition of a force, of a hostility. On the contrary, this is a question of peace-loving opposition, where peace is not the suspension of war, violence momentarily withheld only to be unleashed more effectively, more efficiently. “It is a pacific opposition,” as Levinas explains, “one where peace is not a suspended war or a violence simply contained” (*Ibid*., p. 19). The violence perpetuated consists in eliminating this very opposition, outwitting it, ignoring the face of the other, avoiding the gaze. Violence is perpetuated in spite of the opposition as formulated in the commandment, “You shall not kill” (Exodus, 20, 13) inscribed on the face even before it is expressed in a formula (see Levinas, “Paix et proximité” [49]).

Biblical prescriptions—such as, for example, “love your neighbor as yourself,” “When a stranger soujourns with you in your land, you shall not do him wrong. The stranger who soujourns with you shall be to you as the native among you, and you shall love him as yourself” (Leviticus, 19.18 and 19.33–34)—refer, beyond, or, better, before all organization of the juridical or political order, to an antecedent form of peace, a condition of peace that is not less fundamental, and that consists in the relation to the other as other, to the foreigner, the stranger, the alien that each human being is for every other. Extra-political or pre-political peace, solicitation for another (see “Entretiens,” in [99], p. 104), precedes rational thought, being in the form of an “I,” assertions of the subject, of knowledge, of objectifying consciousness. The situation of peace and responsibility in the relationship with the other, where individuals give themselves in their singularity, difference, non-interchangeability, unreplaceability, non-indifference, precedes politics and logic. Politics and logic share the fact that they consider individuals in their social roles, as belonging to a genre, as equals. But the relation of alterity, absolute alterity, is pre-political and pre-logical.

Primordial peace is the paradoxical and contradictory responsibility for a peace that is foreign [47], it implies an interpersonal relationship in which the subject reaches the dignity of the human condition on “assuming responsibility for the other man in the act of election that raises him up to this height. This election comes from a god—or God—who beholds him in the *face* of the other man, his neighbor, the original ‘site’ of the Revelation” (Levinas, “Preface,” 28 March [4] to the English translation of [35]).

As member of a group I am obliged to keep faith to this responsibility and to relate to every other individual, to every other indiscriminately, therefore in addition to the singularity, I am also obliged to relate to the other of a group, an assemblage, a community who as such is interchangeable with any other individual member of that same group, in this sense indifferent to me. To know, judge, do justice, to confront two individuals in order to identify the guilty one, all this requires generalization through logic and the State, equalization of singularities on the basis of reference to a group, an assemblage, insofar as they are citizens belonging to the same State. The relation to the other is mediated by institutions and by juridical procedure. All this generalizes and at the same time delimits responsibility, the responsibility of each one for every other. From this type of generalization there derives a need for the State (see Levinas, “Entretiens” [99], p. 118).

The function of the State consists in limiting and defining the pre-political and pre-logical condition of absolute otherness, which precedes institution of the State. On Levinas’ account, the State, or State justice, does not found personal responsibility for the other, it is not at the origin of responsibility for the other. On the contrary, State justice limits and defines original responsibility, places boundaries on it, and at once guarantees limited responsibility through generalization of the law, responsibility with alibis.

Instead, unlimited responsibility, responsibility for the other, unconditional, undefined, imperative, absolute, moral responsibility is not written, it is not inscribed in the law. It does not converge with State justice. Indeed, from this point of view State justice always results imperfect with respect to human rights understood as the rights of the other as other, as foreigner. The commitment to human rights is not a function of the State, but rather a non-State institution in the State, an appeal to humanity, yet to be accomplished in the State (see *Ibid*., p. 119).

The limits of individual responsibility, limits of the political, juridical, ethical-normative order, of behaviour regulated by the laws of equal exchange, of functions fixed by roles and social position, in relation to one’s status in the social, distinctions between individual identities sanctioned by the law, identities and differences whose sphere of freedom and imputability is at once limited and guaranteed by the law: none of all this will succeed in undoing the intrigue, the entanglement between the I and the other, in eliminating the inherent asymmetry in the I-other relationship, in impeding obsession for the other, in putting an end to involvement with the other, in avoiding substitution.

## Justice and Proximity

To deal with the problem of justice is to deal with a problem that is recurrent in the history of philosophical thought. The problem of justice is closely connected to that of my responsibility for the other and of the other’s responsibility for me. Consequently, according to Levinas, the problem of justice is essentially the problem of justice in favour of the other, in defence of the other. Therefore, it also involves the question of impeding violence and guaranteeing peace. The problem of the perfectibility of justice also comes to the fore [78]. Levinas associates it to the question of non-indifference towards the other. All this leads Levinas to challenge the traditional understanding of human rights. He evidences the need to reorganize and reinterpret human rights so that they include the rights of the other.

The question that comes to the attention immediately and is proposed repeatedly is the following: whether responsibility for others is a consequence of the law established by the State or whether the State with all its laws arises to guarantee my responsibility not only for the other close to me, my neighbour in a spatial sense, but for every other, and responsibility of every other for me (see “Responsabilité et substitution,” in Ponzio, *Sujet et alterité* [100], pp. 144–146). The law establishes that I must concern myself not only with my “neighbour” in the sense of spatial vicinity, not only with the “first comer,” but also the second, the third, and all others. Each I is an other. The exclusive nature of the relationship of an I to his neighbour is modified. This is the situation that gives rise to “justice”. Recourse to justice, the invention of justice, the formation of courts and judges arise, in effect, as Levinas says, from the fact that my relationship to the other is not limited to *one* only, or to any other single individual other, but rather implies *many* others, indeed even all others. The question that arises is why practice compassion, mercy towards one and not the other, why care for one and not the other, even when the other would never do the same for me, does not care for me. It ensues that there is a need for rules, for laws in order to equate what cannot be equated, the singularity, the uniqueness of each one.

The consequence of “justice” is that each one must be equal in front of the law, all equal before the law, so that the singularity of each, the uniqueness of each one paradoxically becomes comparable to the singularity, the uniqueness of every other. On this account Levinas speaks of “comparison among incomparables,” in other words, comparison among singularities, among the uniqueness of each one. Extension of responsibility of the I in front of the other, of all the citizens of a State, involves their “equalization” (cf. [106]). Justice arises for this, and may subsist only on the condition that, in the last analysis laws, the State, comparison among incomparables, among singularities have at their foundation responsibility for all others, and not only for some, even those most distant, and not only for those who are closest.

From this point of view, proximity (my “neighbour”), as we have already said, from a concept of the spatial order turns into a concept of the ethical order and becomes responsibility. My neighbour, close or distant as s/he may be in space, is the person for whom I am responsible, whoever s/he might be. And justice, laws, the State arise as a function of this irreducible relationship of responsibility, on the basis of this relationship of responsibility. Even if it is circumscribed and regulated, the responsibility attributed to each one, according to Levinas, is the positive aspect of the constitution of laws. And in this origin, in this motivation for the formation of laws and the State, there is also an implicit indication that justice and the laws that govern it are always, and always again, perfectible, they can always be improved.

The law is a function of justice, for the sake of justice, and in the conditions described, justice is never definitive, it cannot be achieved once and for all. Justice foresees its own continuously problematic nature, the ongoing possibility of its very own improvement, justice involves *problematization of the law*, the *perfectibility of laws*. Therefore, differently to the claim made by Thomas Hobbes, responsibility for the other does not arise as a result of the formation of the State, of the constitution of laws and of justice, but, on the contrary: it is on the basis of original responsibility for the other, and of the opportunity, for reasons of justice, of its extensibility to every other, that States and laws are born. And if we must resort to the condition of “fear” to explain the formation of the State, this is not at all a question of “fear of the other” caused by a presumed original situation of *homo homini lupus*, as Hobbes recounts, but of *fear for the other*, of concern for the other, the original situation of non-indifference for the other in which the I as such comes to find itself. Thus with Levinas the Hobbesian conception of *homo homini lupus* is at last inverted: at the origin of the State is not fear *of* the other (as Hobbes recounts), but fear *for* the other (Levinas 1998; “Ideology and idealism” [55], p. 247, cf. Levinas [99], pp. 104–105 and 115–119).

From this point of view, the original relation of responsibility of the I in front of the other is regulated, defined with the State and its laws: if I must be responsible not only for this other, but for every other, responsibility must necessarily be re-dimensioned and delimited, establishing precise conditions and situations within which we are responsible. So then, the birth of the State and its laws does not found responsibility for others, but delimits it. Uniqueness of the I and of the other is traced back to the individual as belonging to a multiplicity, a community, and responsibility here is relative to identity, established through belonging to a collectivity, a group, a role, a profession, even a given age.

Politics and justice are necessary in the constitution of the State. However, politics and justice are appealed to, challenged by mercy, as results from the concern to recognize every other beyond one’s own neighbourhood. The State does not found personal responsibility towards the other, but limits and defines it, though it guarantees responsibility through generalization of the law. Instead, unconditional responsibility, which concerns the individual in its singularity, does not converge with State justice. In this respect State justice is always imperfect when a question of human rights understood as the rights of the other, the foreigner, the outsider (*Ibid*. pp. 118–119).

That the State, the liberal State, the State in democracy can review its justice and its laws with the intention of improving them, that justice and its laws can be perfected signs the primordial propensity of the I for the other at their origin, to the benefit of all singularities. Perfectibility and renewal cannot be achieved simply through a process of logical deduction from a doctrine intended to be ever more precise. When the State appeals to invariable justice, justice that is logically deduced, what it is announcing is the rise of the totalitarian State. On the contrary, at the foundation perfectibility and renewal is a commitment of the moral order, of the ethical order, in our terminology the *semioethical* order.

## Literary Writing

Another concept which characterizes the research of Levinas is that of “*écriture avant la letter*” (“writing before the letter”), which A. Ponzio invites him to discuss towards the conclusion to their conversation of 1988. Levinas critiques the idea of the priority of literal meaning and keeping account of the sense of literary writing maintains, instead, that the word refers laterally to other words: “Ils [les mots] ne seraient pas figés dans un sens littéral. Il n’y auraient d’ailleurs pas de sens littéral. Les mots ne renverraient pas à des contenus qu’ils désigneraient mais en premier lieu, latéralement, à d’autres mots” (*Humanisme de l’autre homme* [42], p. 20). The image of the word referring laterally, from the corner of the eye we might say, to other words, leads us to think of the word in the novel as “indirect speaking,” a concept described by Mikhail Bakhtin (see “Discourse in the Novel,” in Bakhtin, *The Dialogic Imagination*, 1984[1934–35], pp. 259–422; “The Problem of the Text in Linguistics, Philology, and the Human Sciences,” and “From Notes Made in 1970–1971,” in Bakhtin, *Speech Genres and Other Essays*, 1986[1959–1961], pp. 103–131 and 132–158). This image of the word referring laterally to other words also leads us to Italo Calvino [25] and his Perseus, the mythological hero who vanquishes Medusa thanks to his “indirect gaze,” just as it evokes the concepts of “intransitive writing” and “obtuse sense” as thematized by Roland Barthes [23]. By referring laterally to other words, with the indirect gaze, the word escapes petrification by the literal word, by reality. Levinas evidences the metaphorical nature of language as an essential characteristic of language, “la metaphoricité essentielle du langage”: because of “metaphoricity,” because of the essential metaphorical natural of language he claims that there is no literal sense (see Levinas, *L’au-delà du verset*, 1982), being a central theme in Victoria Welby’s writings (see Petrilli 2009, pp. 351–363; [76],and Welby [1893], in *Ibid*. pp. 421–439; [121] [1879–1911]; [122] [1879–1911]). This infinite deferral of meaning and of experience that the metaphorization of signifying processes implies has consequences on how we conceive reality. In this situation of continuous deferral, of shift in sense, experience itself becomes reading, exegesis, hermeneutics, so that literary value too assumes truth value on the methodological and exegetical level (see [71], pp. 191–230).

Literary writing shows how the meaning of words is never definitive, closed, finalized, and never limited to our everyday needs. Writing, the literary word, is not restricted to the transmission of information, to the pragmatic functionality of the day. Exegesis is in continuous expansion, it renews the sense of the written text, “elevates it to the truth”. This is writing that challenges and interrogates the parallelism established by Edmund Husserl between the noetic and the noematic. As though writing, poetics in its essence is dictated from the outside; writing beyond knowledge. Writers, sacred and profane, authors of literary writing (to exemplify, Levinas evokes Rimbaud, Baudelaire, Goethe, Shakespeare, Dante, Racine, Corneille and Molière) reveal a philosophical consistency (Levinas, *Humanisme de l’autre homme* [42], p. 21). These writers testify to the capacity of sense, of meaning to surpass the elements that were thought to enclose them, to entrap them according to logics of the identity order. All this means that the vocation of literary writing, of artistic discourse in general, whether sacred or profane, is that of transcending the limits of knowledge and of reason.

In Levinas the sense of responsibility towards the other has no limits to the point that if the other is guilty for something, I too am involved in this guilt, to the point even of being more guilty than that other. Like Bakhtin, Levinas read Dostoevsky and evoking a character from *The Brothers Karamazov,* he maintains that we are all guilty, but I myself more than anybody else. Like Levinas, Dostoevsky, a master in literary writing, traces the “original constitution” of the I, of singularity, in responsibility for one’s neighbour, the other, and in impossible withdrawal from such responsibility, in the impossibility of being replaced by anybody else. I am responsible in an absolute sense: like a hostage I must answer for something I did not do, for a past never mine, never present to me, as says Levinas [99], p. 118.

It is precisely this situation of irreplaceability of each one in the face of the other that renders the each one singular, unique. To the I’s uniqueness there corresponds the other’s uniqueness, the other’s face as other, and the uniqueness of responsibility occasioned for me by that other. I and other in their singularity are not just individuals that belong to the same genus, to the same abstract class, as anonymous members in a logically extended concept. The relation to the other as other, to the other in that other’s otherness, “*autrui*”, is irreducible to subjective experience, to the truth of knowledge, cognition and representation.

Recognition of the other as other implies the primacy of ethics, not ontology, in human understanding, love for the other, which does not begin in the erotic, love without concupiscence. The “beloved”, the “loved one” is unique, the only one in the world for the “lover”, the “enamoured”—love, the dis-inter-ested love of responsibility, love in responsibility, love as charity, mercy and care for the other. Impossible escape from responsibility is not passive slavery, but election, and as world religions teach us “election” is indicative of the supreme dignity of the human, of human worth for its own sake, as the thing in itself (cf. [3], pp. xii–xv). Therefore, contrary to Hobbes when he maintains that justice and the State arise from the original situation of *homo homini lupus*, for Levinas at the beginning of the State is charity, love for the other; justice and the just State are no less than the way to charity in the human multiplicity. In Levinas, the State must place limitations on charity, but despite this, and despite the fact that laws and State justice are liable to perfectibility as a result of such limitations, the State and its justice is at once anchored in love.

Levinas ends his conversation of 1988 with the expression of a doubt [102], pp. 147–148: “Je me suis souvent demandé si le commencement de la vérité, la première vérité cartésienne—le *cogito*—avant toutes les chances qu’il renferme de ramener un jour à Dieu—n’est pas déjà prière criée du fond d’une solitude de doute” (“I have frequently asked myself if the beginning of truth, the first Cartesian truth—the *cogito*, before all the possibilities it contains of leading one day to God—is not already prayer cried from the depth of a solitude of doubt”).

## Unjust Justice

To the extent that the Levinasian notions of “proximity,” “responsibility” and “substitution” do not enter the categories of knowledge, they emerge as *ethical* categories. In other words, these notions are not reducible to knowledge and ontology. In the language of Levinas “proximity,” “responsibility” and “substitution” can be read as a string of words where the subsequent word acts as interpretant of the word that precedes it. In this string, even if of the three notions the ethical dimension is most evident with the notion of “responsibility,” all three assume ethical value and as such are differentiated from ordinary usage where they are associated with awareness, consciousness, standpoint, choice. Contrary to the conscious subject, the subject that is ready to answer for itself, whose behavior is characteriszed by the will to deliberation, by intention, volition, in Levinas’s view the ethical dimension of language says of the condition of non-indifference, of unintentional and inevitable involvement with the other, of human responsibility that is undecided, unforeseen, unplanned. Hence, in Levinas’s vision of the world they tell of a situation of infinite, ongoing concern for the other, in Italian rendered effectively with the expression “essere in pensiero per l’altro,” literally “being in thought for the other”: not thinking the other, not objectifying the other in the subject-object relationship, but thinking of the other, being concerned for the other; not fearing the other, but fearing for the other, in a situation marked by a bad conscience provoked by the other who allows for no respite, no truce. This is what is implied by the ethical dimension of sign and language.

Justice regulates social relations and is founded on *ethics* thus conceived, oriented by value systems that can vary from one culture to another.

But the globalized world population responds to the dominant ideology of Occidental reason, where society and the individuals that compose it are leveled onto equal exchange market values. Under this aspect globalized society recalls socialist society as described by Levinas in “Trascendence et hauteur” [40]: though intended to free the I from alienation provoked by injustice committed by the I itself, real socialism continues to be engaged in recognizing the rights of the I, it does not stop signifying its being for the I. As Levinas claims, man is conceived as an I or as a citizen, never in the irreducible originality of his alterity which cannot be accessed in reciprocity or symmetry. Egalitarian law and universality produce conflicts that put primitive egoisms into relationships of opposition (*ibid*., p. 103). These reflections by Levinas can be related to Marx’s critique of real socialism *ante litteram*, what in “Private property and communism” [60] he defines as “crude” and “thoughtless” communism. Marx refers here to the tendency to suppress private property, generalizing it and extending it to all, thereby promoting the whole community to the status of capitalist, characterized by envy and the tendency to leveling-down, by abstract negation of the entire world of culture and civilization, and by a strong will to deny talent.

In the context of globalization, it is difficult more than ever to keep account of Levinasian ethics of fear for the other, of unlimited responsibility for the other, of unindifference towards the other whom, on the contrary, the I often abhors and despises. Against unjust justice in the United States of America, particularly in response to injustices perpetrated by the ideology of white identity, *white supremacy*, in the face of people of colour (Afro-Americans, South Americans, Asians, etc.), a new discipline has emerged in the juridical field and in the context of the debate about civil rights and social justice to introduce the rights of the other in human rights as applied by legal institutions. This new discipline has been denominated “critical legal studies” and is connected to “critical race theory” (see Athanor, *White Matters. Il bianco in questione*, [8], Petrilli, *Un mondo di segni*, 2012, pp. 107–113; *Challenges to Living Together*, [3], pp. 41–42).

In a society that is taking shape ever more in terms of multiculturality and plurilinguism we are urged to overcome the limits of legality characteristic of the legislative State with its prejudices and stereotypes and render justice less unjust, thereby introducing elements of justice into the structure itself of juridical discourse (see Athanor, *Fedi, credenze, fanatismo*, [13], Athanor, *Pace, pacifismo e i loro linguaggi* [14]).

With Levinas this means to recover the primordial movement towards the other at the origin of the sign, of language and of social justice, the condition of *responsibility* that is also *responsiveness* towards the other, fear for the other before and beyond identity itself, and thus reestablish the connection between legality and justice, human rights and ethics as understood by Levinas.

## Caring for the Other

Human life over the entire planet is hostage to a virus, Sars-2, cause of the current pandemic, from the Greek, “*pandemos*” (“*pan*” = “all”, “*demos* = “people”), Covid-19, threatening the world population in its totality. And if the goal is to counteract illness and death and safeguard all peoples of the earth, it seems that immunity must be just as total. Nor will it suffice to produce the vaccine, it must also be allowed to circulate. And here indifference towards one’s neighbour in our globalized world is manifest. The virus Sars-2 has contributed to unmasking the inhuman nature of the human, to revealing and at once exasperating social diseases and their symptoms (see [67]) economic precarity, racism, classism, misogeny, homophobia, xenophobia, all sorts of inequalities and social injustices, endemic to the presentday globalized economico-social system. But not even this virus, cause of the illness Covid-19, moves alone, it too flourishes in the interspecific dialogue with human life. And in the interface between human and nonhuman, Sars-2 fully manifests itself as a cultural, socio-economic phenomenon, itself a symptom of socio-economic and ecological pandemics structural to globalization: the gap between wealth and poverty, development and underdevelopment, the rhythm of technological progress and death through hardship, between an infant born into a well-off family and the slaughter of infants who continue dying from famine and sicknesses now eradicated from the “developed” world, the invisibile children, the gap between employment and unemployment, between those who have refuge, a home and those who do not, who are forced to migrate, all of which generated and exacerbated in the infinite dialectics between war and peace—that is the peace of war (Petrill and Ponzio, “Il diritto alla pace e la globalizzazione della guerra infinita,” in Petrilli [83], pp. 419–454.

Just as food does not circulate for all—and the same for water, the situation is no different for the question of immunization. Health and sickness are plainly manifest in their ideological nature, as organico-socio-economic phenomena: social injustice in globalization, itself a “viral” disease. From a global viewpoint, anti Covid-19 immunity over the planet is still scarce both in terms of production of vaccines and of their circulation, a fact that—in light of Levinas’s analyses of the human condition in terms of primordial fear for the other, responsibility towards the other antecedent to politics and logic, of the original propensity for care of the other—interrogates world social justice and demands ethical commitment beyond economic market reason and the ideology of globalism.

Never before, in a globalized world closed in upon its own identity, on short-sighted self-interest, is it so urgent to listen to a voice like that of Emmanuel Levinas, thus overcome the walls of silence and indifference towards the other, and of greed. Levinas teaches us that the universal is constructed in dialogue with the particular, the singular, the unique, a dialogue that language presupposes and that just justice must recover. Language for Levinas is proximity with respect to the other, responsibility, substitution, its cypher is the absolute other, in sociality un-self-interested love and care for the other. And as global semiotics teaches us—which as global semiotics evidences the inexorable condition of interconnection and interdependency among all life-forms in the biosphere (that coincides with the semiosphere)—semiosis, therefore the health of life generally and the possibility of projection towards a better future, presupposes the health of each single individual: if the whole community is to stay healthy, it will be necessary to care for the health of each one. Giacomo Leopardi had already made the point when in a letter to Pietro Giordani he asked the simple question of whether the happiness of entire popoulations can be achieved without the happiness of individuals (*Zibaldone*, Florence, 24 July 1828).

With Emmanuel Levinas reread in light of global semiotics, we know that values resound throughout the network of signs and of life in its totality. Semiotic materialism evidences how beyond converging with life, signs in the human world carry ideas, visions of the world, ideologies. The implication of the I in the life of others is a given as the pandemic of globalization has contributed to evidencing, where “of globalization” resounds both as an object genitive and as a subject genitive, such that in Levinas’s terminology, the “ethical intrigue” is ever more clearly inescapable, referring to a social situation in which individuals are valued in their singularity, difference, non-interchangeability, unindifference, eachness, and where the single individual is implicated, bridled, in spite of oneself, in the condition of responsibility towards the other, impossible to delegate, as Mikhail Bakhtin avers (see [80, 81, 85]). If the common goal is the health of life, of *life overall human and nonhuman*, *of semiosis at the planetary level,* beyond the walls of indifference, of short-sighted identity ideologies, closed and greedy, it has become ever more urgent to recover the primordial propensity for otherness, *love for the other at the origin of language and communication, of knowledge and legal systems*, and practice social justice in close dialogue with the other perceived, experienced and welcomed in that other’s uniqueness as other.
